# Suicide risk in caregivers of people with dementia: a systematic review and meta-analysis

**DOI:** 10.1007/s40520-022-02160-6

**Published:** 2022-06-13

**Authors:** Luisa Solimando, Marta Fasulo, Stefano Cavallero, Nicola Veronese, Lee Smith, Laura Vernuccio, Francesco Bolzetta, Ligia J. Dominguez, Mario Barbagallo

**Affiliations:** 1 Geriatric Unit, Department of Internal Medicine and Geriatrics, University of Palermo, Via del Vespro 141, 90127 Palermo, Italy; 2 Centre for Health Performance and Wellbeing, Anglia Ruskin University, Cambridge, CB1 1PT UK; 3 Medical Department, Geriatric Unit, Azienda ULSS (Unità Locale Socio Sanitaria) 3 “Serenissima”, Dolo-Mirano District, Italy; 4 Faculty of Medicine and Surgery, University of Enna “Kore”, Enna, Italy

**Keywords:** Dementia, Caregiving, Suicide, Meta-analysis, Alzheimer

## Abstract

**Background:**

Interest in physical and mental health outcomes in caregivers of patients with dementia is increasing. However, there is limited data available on the prevalence of suicidal ideation, suicidal attempts, and suicide rates among caregivers of those with dementia. Therefore, we aimed to systematically review these outcomes to address this gap in the literature and thus provide information to inform future policy and intervention for the benefit of caregivers of dementia patients.

**Methods:**

We searched several databases from inception to the 10^th^ November 2021, for studies investigating suicidal ideation, suicidal attempts, and suicide rates of caregivers of patients with dementia. We report data regarding suicidal ideation as prevalence, with the 95% confidence intervals (CIs), applying a random-effect model; since less than three studies were available for suicide attempt and suicide, these data are reported descriptively.

**Results:**

Among 194 articles, eight comprising 1,209 informal caregivers of people with dementia (mean age: 63.9 years, 74% females) were included. The prevalence of suicide ideation was 32.32% (95% CI: 16.01–48.64%; *I*^2^ = 98.5%, *p* < 0.0001). The prevalence of suicide ideation varied between studies from 4.69% to 77.78%. Two studies reported the rate of suicidal attempt in caregivers of patients with dementia, with the prevalence ranging from 5.9% to 16.1%. One study reported one in 17 caregivers of patients with dementia died by suicide.

**Conclusions:**

The prevalence of suicide ideation is high, affecting several caregivers of patients with dementia. These findings suggest intervention and/or policy are urgently needed to address suicidal behavior in this at-risk population.

**Supplementary Information:**

The online version contains supplementary material available at 10.1007/s40520-022-02160-6.

## Introduction

The number of people living with dementia worldwide in 2015 was estimated at 47.5 million, reaching 75.6 million in 2030 [1]. Future projections indicate that this number will be 135.46 million in 2050 [1]. Approximately 7.7 million new cases of dementia are anticipated each year [1]. In the US, costs attributable to their care range between 157 and 215 billion dollars and are expected to more than double by 2040 [2]. Moreover, the cost linked to informal caregiving in older people with dementia are difficult to estimate, but it is likely to be overall increasing.

Family members are the main careers for people with dementia living at home in almost all countries. It has been widely reported that the caregivers of patients with dementia have high levels of stress and high rates of depression and anxiety [3]. Therefore, often experiencing worse physical health than non-caregivers. Indeed, all these factors are potential risk factors for suicidal behavior [4]. It is largely known that caregivers of people with dementia witness a progressive decline in the person’s cognitive capacity, communication skills, and functional abilities, and thus learn new skills to manage behavioral and psychological symptoms common in dementia [5]. These daily problems may contribute to a range of negative physical and mental health outcomes [6], with a consequent increase in caregiver’s morbidity and mortality [7].

In recent years, there has been a rapid increase in scientific literature examining caregiver’s health and the difficulties encountered in caring for people with dementia. In addition, literature relating to suicidal behavior among caregivers of those with dementia is now emerging but to date no attempt has been made to collate, synthesis, and understand this literature as a whole. Given this background, we aimed to systematically review the prevalence of suicidal ideation, suicidal attempt, and suicide rates in caregivers of patients with dementia to address this gap in the literature and thus provide important information to inform future policy and intervention for the benefit of caregivers of dementia patients.

## Materials and methods

This systematic review adhered to the PRISMA statement [8] and followed a pre-planned protocol that can be requested by contacting the corresponding author.

### Data sources and searches

Four investigators (LS, MF, SC, NV) independently conducted a literature search using Web of Science, Pubmed/Medline, and PsycINFO from database inception until 10 November 2021.

In PubMed/Medline the following search strategy was used: “(Caregiver* OR Carer* OR Care Giver) AND (dement* OR alzheimer* OR Lewy OR “Posterior cortical atrophy” OR Binswanger OR “Progressive supranuclear palsy” OR Frontotemporal disorder* OR Frontotemporal degeneration OR Corticobasal degeneration OR Corticobasal syndrome) AND (suicide OR Suicidal Ideation OR Ideation, Suicidal [mh])”, adapting the search for the other databases. Any inconsistencies during title, abstract and finally full-text screening were resolved by consensus with a third senior author (LJD).

### Study selection

Inclusion criteria for this systematic review were: (i) presence of informal caregiving, i.e., persons who provide some type of unpaid, ongoing assistance with activities of daily living or instrumental activities of daily living to a person with a chronic illness or disability [9]; (ii) report data regarding suicide or suicidal ideation or suicidal attempt; (iii) presence of patients with dementia. We excluded: (i) papers not relating to patients with dementia; (ii) data regarding suicidal behavior in patients with dementia per se.

### Data extraction

Three independent investigators (LS, MF, SC) extracted key data from the included articles in a standardized Excel spreadsheet, with another independent investigator (NV) checking the data. For each article, we extracted data on authors’ name, year of publication, country, sample size, mean age and percentage of female caregivers, type of dementia, how suicide/suicidal ideation/suicidal attempt were defined, and tools for identifying suicide attempt/ideation.

### Outcomes

The primary outcome of our systematic review was the prevalence of suicidal ideation in informal caregivers of people affected by dementia. Suicidal attempt and (completed) suicide were considered as co-primary outcomes.

### Data synthesis and analysis

The primary analysis compared the overall prevalence of suicide ideation in caregivers of people with dementia. We, therefore, report this information as prevalence, weighted for sample sizes with the 95% confidence intervals (CIs), applying a random-effect model [10]. Since less than three studies were available for suicide attempt and complete suicide, these data are reported descriptively.

Heterogeneity across studies was assessed by the *I*^2^ metric and χ^2^ statistics. Given significant heterogeneity (*I*^2^ ≥ 50%, *p* < 0.05) and for outcomes having at least ten studies, we conducted a series of meta-regression analyses [11], using as moderators mean age, percentage of female caregivers and type of dementia. Publication bias was assessed by visually inspecting funnel plots and using the Egger bias test [12] and in case of publication bias a fill and trim analysis was performed [13].

For all analyses, a P-value less than 0.05 was considered statistically significant. All analyses were performed using STATA version 14.0 (StataCorp).

## Results

### Literature search

**Fig. 1:**
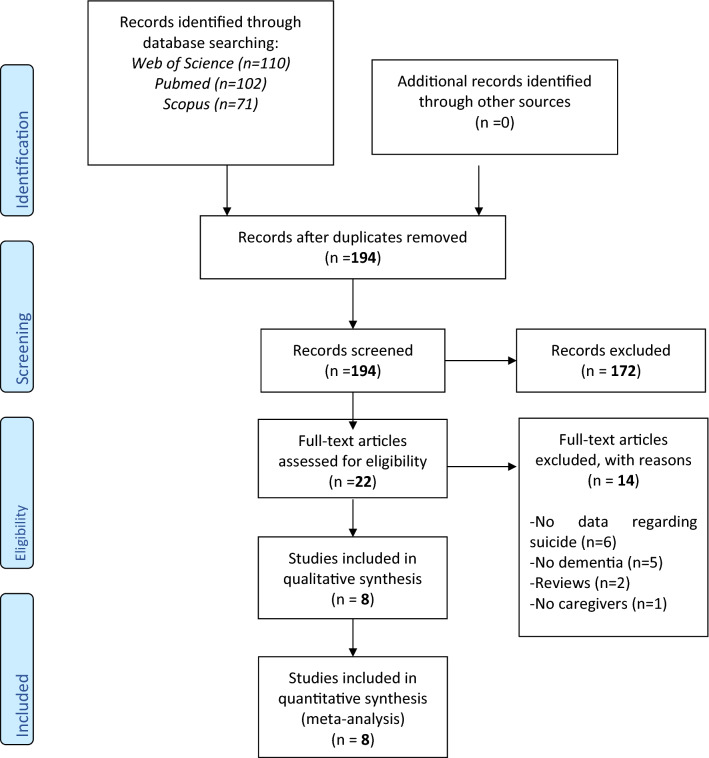
PRISMA flow-chart

As shown in Fig. 1, we initially screened 194 articles. After removing 172 records, based on the tile and the abstract, 22 full texts were examined and eight articles were finally included [14–21].

### Descriptive characteristics

As reported in Table 1, 1209 informal caregivers of people affected by dementia were included. Caregivers had a mean age of 63.9 years and were mainly female (74%). Two studies included only patients with Alzheimer’s disease [14, 20], one study with either Alzheimer’s disease or fronto-temporal dementia [16], whilst the other five did not specify syndrome type. The tools used for identifying suicidal ideation or attempt varied across studies (full details in Table 1).

**Table 1:** Descriptive characteristics of the studies included

Author	Year	Country	Sample size	Mean age	% of females	Type of dementia	Suicide definition	Tool for identifying suicide ideations or attempts
Anderson	2019	UK, USA	9	Not available	88.8	AD	Not specified	Blogs written by caregivers
Joling	2018	Netherlands	192	70	70	Not specified	Not specified	Mini international neuropsychiatric interview
Koyama	2017	Japan	104	Not available	58	Not specified	Not specified	Self-reported
Parkinson	2018	Netherlands	192	69.5	70.3	Not specified	Not specified	Mini international neuropsychiatric interview
Shaji	2003	India	17	Not available	76	AD	Reported by family members	Self-reported
O’Dwyer	2016	Australia	566	62.93	84	Not specified	Not specified	Suicidal behaviors questionnaire-revised
O’Dwyer	2013	Australia	9	58.33	55	Not specified	Not specified	Non-structured interview about their caring experience
O’Dwyer	2013	Australia	120	58.76	89.6	AD and FTD	Not specified	Six online career discussion boards hosted by Australian, US and UK
Total			1209	63.9	74	AD (*n* = 2); AD and FTD (*n* = 1); not specified (*n* = 5)	Reported by family members (*n* = 1); not specified (*n* = 8)	

*AD* Alzheimer’s disease; *FTD* fronto-temporal dementia

### Informal caregiving and suicide

**Fig. 2:**
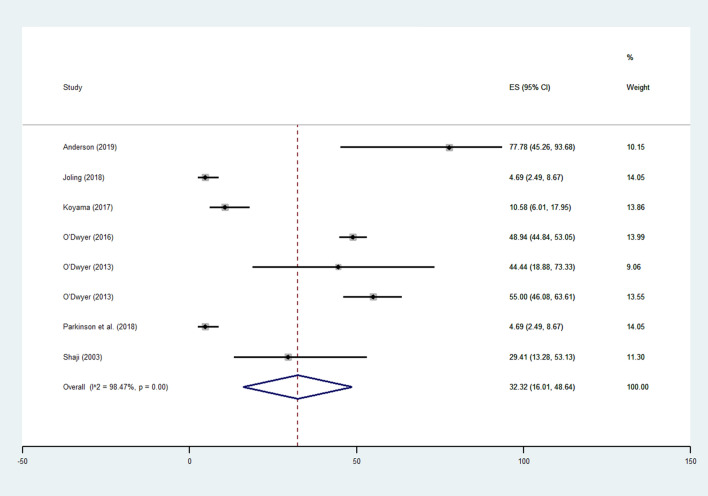
Prevalence of suicide ideations in informal caregivers of patients affected by dementia

As shown in Fig. 2, all studies reported data on suicidal ideation. Overall, the prevalence of suicidal ideation in the 1209 caregivers included was 32.32% (95% CI: 16.01–48.64%) characterized by a high heterogeneity (*I*^2^ = 98.5%, *p* < 0.0001). The prevalence of suicidal ideation varied between the studied from 4.69% [19] to 77.78% [20]. This outcome did not suffer from any publication bias, based on the Egger’s test (5.68 ± 4.67; *p* = 0.27) and the visual inspection of the funnel plot.

Since the prevalence of suicidal ideation was characterized by high heterogeneity, we performed several meta-regression analyses showing that a higher mean age was associated with a lower prevalence of suicidal ideation: for each one-year increase in mean age, the rate of suicidal ideation decreased by approximately 5% (beta =  − 0.05; 95% CI: − 0.07 to − 0.02; *p* = 0.009; *R*^2^ = 91.7%). Moreover, when stratifying the data by type of dementia, no significant differences across strata emerged (*p* = 0.34) and the heterogeneity (described by the *I*^2^) remained more than 50% in all the strata.

Two studies reported the rate of suicide attempt in caregivers of patients with dementia. One study performed on 566 caregivers [17], reported a prevalence of suicide attempt of 16.1%, whilst the other study 5.9% [14].

Finally, one study [14] reported one in 17 caregivers of patients with dementia died by suicide.

## Discussion

In this systematic review and meta-analysis using eight studies including 1,209 informal caregivers of people with dementia, a high prevalence of suicidal ideation was present, approximately one in three informal caregivers.

Our data showed that the prevalence of suicidal ideation is more evident in younger compared to older caregivers; however, the analyzed data only concerned caregivers, without comparing the rate of suicidal ideation with the data of the general population. Therefore, it would be important to investigate the prevalence of these conditions with population-based studies. In this way, it would be possible to utilize more accurate data on risk of suicidal ideation in the interested population. In addition, it would be relevant to value how these results change according to the country where the study was conducted. Indeed, we observed that the prevalence of suicidal ideation varied by country where the study was conducted. In a study carried out in Japan, in contrast to the other studies included, an increase in suicidal ideation was not observed, likely owing to differences in cultural background [18]. Unfortunately, in Japan, the rate of suicide and suicidal ideation is high in the general population, potentially explaining the non-significant results [22]. In addition, in this study, in contrast to the others, depression was not a risk factor for suicidal ideation [18]. Finally, only two studies reported data regarding suicide attempt indicating that prevalence is high.

Several studies demonstrated that specific factors may influence the way in which family members address caregiving, e.g., caregiver’s sense of competence. This is defined as a state related to the caregiver’s feeling of being capable to manage the caregiving role [23]. A positive sense of competence has been associated with a reduced risk of the person with dementia being institutionalized [24]. Approximately 80% of people with dementia are cared for by their family and they are often institutionalized after 6.5 years, as demonstrated by several studies [25]. One potential reason for institutionalization may be owing to the presence of depression in patients, which is also associated with lower quality of life in caregivers, combined these lower the self-sufficiency of demented patients [25]. Moreover, reduction of activities of daily living may result in a greater level of care that consequently increases caregiver’s stress. Caregiver depression is thought to be a consequence of care due to a complex interplay of factors that comprises dimensions of the patient, caregiver, and cultural background [26]. It is important to note that a potential increase in suicidal behavior is associated with all caregivers and not just caregivers of those with dementia. For example, a large study has demonstrated that suicide rates are twice as high as that of the general population among caregivers of patients with cancer [27]. An important risk factor, as also described in our systematic review, is depression, even though other risk factors (such as psychiatric history, previous suicide attempts, hopelessness, demoralization, pain, lack of social support, feeling of being a burden to others, and existential concerns) also may confer increased suicidality [28].

The findings of our study must be interpreted within its limitations. First, the number of studies and participants are limited, prohibiting some analyses. Another limitation is represented by the difference in tools used across these studies to identify suicidal ideation, often self-reported during interviews. Moreover, the prevalence of suicide and suicidal ideation in caregivers of those with dementia was not compared with general population data. Finally, we were not able to describe the specificity in the definition of suicidal ideation (i.e., active or passive or intermittently), but this topic is of critical importance and merits further research.

In conclusion, our systematic review and meta-analysis reported that the prevalence of suicidal ideation is high, affecting a high proportion of caregivers of patients with dementia. Since the topic of suicide is often not discussed in daily clinical practice, we encourage further research of a qualitative nature to further inform intervention and policy on suicide reduction among caregivers of those with dementia.

## Supplementary Information

Below is the link to the electronic supplementary material.

Supplementary file1 (DOCX 31 KB)
